# Fewer native and periprosthetic femoral fracture patients receive an orthogeriatric review and expedited surgery compared to hip fracture patients

**DOI:** 10.1177/11207000231198459

**Published:** 2023-09-18

**Authors:** Muhamed M Farhan-Alanie, Sam C Jonas, Daniel Gallacher, Michael R Whitehouse, Tim JS Chesser

**Affiliations:** 1 Warwick Medical School, University of Warwick, Coventry, CV4 7HL, UK; 2 Cardiff and Vale Orthopaedic Centre, University Hospital Llandough, Penarth, UK; 3 Department of Trauma and Orthopaedics, North Bristol NHS Trust, Southmead Hospital, Bristol, UK; 4 National Institute for Health Research Bristol Biomedical Research Centre, University Hospitals Bristol, Bristol, UK; 5 Musculoskeletal Research Unit, Translational Health Sciences, Bristol Medical School, University of Bristol, UK

**Keywords:** Femoral fracture, hip fracture, management, periprosthetic hip fracture

## Abstract

**Introduction:**

Disproportionate emphasis has been attributed to hip fracture over other femoral fractures through implementation of Best Practice Tariff (BPT).

This retrospective comparative observational cohort study aimed to evaluate the epidemiology of native and periprosthetic femoral fractures and establish any disparities in their management relative to hip fractures.

**Methods:**

All patients ⩾60 years admitted with a native or periprosthetic femoral fracture during July 2016–June 2018 were identified using our hospital database. Results were compared to National Hip Fracture Database data over the same period.

**Results:**

58 native femoral, 87 periprosthetic and 1032 hip fractures were identified. (46/58) 79% and 76/87 (89%) of native and periprosthetic femoral fractures were managed operatively. Surgery was performed <36 hours for 34/46 (74%) of native femoral and 33/76 (43%) of periprosthetic fractures compared to 826/1032 (80%) for hips. Median time to surgery was longer in periprosthetic femoral than hip fracture patients (44.7 vs. 21.6 hours; *p* *<* 0.0001). Orthogeriatrician review occurred in 24/58 (41%) and 48/87 (55%) of native and periprosthetic fractures compared to 1017/1032 (99%) for hips (*p* *<* 0.0001). One year mortality was 35%, 20% and 26% for native femoral, periprosthetic and hip fracture patients. Cox proportional hazard ratio was higher for native femoral than hip fracture patients (1.75; 95% CI, 1.12–2.73).

**Conclusions:**

This study demonstrates large disparities in management of other femoral and periprosthetic fractures compared to hip fractures, specifically time to surgery and orthogeriatrician review. This may have resulted in the comparatively higher mortality rate of native femoral fracture patients. Expansion of the BPT to include the whole femur is likely to improve outcomes.

## Introduction

In recent years, there has been a focus on improving the care of elderly patients sustaining fragility hip fractures, concentrating on rapid assessment, optimisation for surgery, early surgery and rehabilitation.^1^ This has largely been driven by the introduction of the National Hip Fracture Database (NHFD) and a pay-for-performance initiative termed the Best Practice Tariff (BPT). The latter incentivises hospitals to achieve six clinical standards for hip fracture patients aged 60 years and older. These include surgery within 36 hours of hospital arrival and assessment by an orthogeriatrician within 72 hours of admission.^2,3^ These approaches have led to significant improvements in outcome for this patient group.^1,4–6^ However, one of the criticisms has been patients who sustain fractures in other areas of the same bone or fractures elsewhere may not get the same treatment and priority.^7^ For fractures involving the shaft and distal femur, and for periprosthetic femoral fractures, there is little data of the incidence and the type of injury and treatment provided.^8^ It has been reported there is a rise in periprosthetic fractures with an increasing incidence and prevalence of patients with hip and/or knee replacements.^9–11^

The dichotomised management of fracture care between hip fracture patients and those who sustain other femoral fractures has led to disparities of care. However, both patient groups require similar complex multidisciplinary care assessment and pathways, sharing similar complications related to recumbency.^1,7,12^

This retrospective, comparative, observational cohort study aimed to:

(1) Evaluate the epidemiology and clinical features of native bone and periprosthetic femoral fractures in a single UK Major Trauma Centre;
(2) Establish time to surgery and length of hospital stay;
(3) Determine rates of complications and mortality;
(4) Assess disparities in the management of this group of patients relative to hip fracture patients treated during the same period.

## Methods

### Patient identification

An electronic patient management system (Bluespier Patient Manager [version 9.2.14. Droitwich, England) was used to identify patients who sustained a native diaphyseal or distal femoral fracture or periprosthetic fracture involving any part of the femur between July 2016 and June 2018 (inclusive) at our institution.

### Eligibility criteria

Minimum patient age was 60 years in accordance with NHFD inclusion criteria.^1^ All consecutive cases admitted to hospital were included and minimum patient follow-up was 1 year. Peri-implant fractures around fixation devices were not included. In the case of patients with bilateral injuries (3 native femoral fracture patients and 1 periprosthetic femoral fracture patient), a single bilateral event was analysed, with further details coming from the first recorded fracture to avoid double counting of exposures and events for analyses such as mortality.

### Data collection

A predesigned data collection form was used for data extraction. Data on a variety of parameters were collected including those comprising the NHFD dataset (V11). The full list of parameters data were collected on is presented in Supplemental Table 1. Inpatient hospital notes and charts, inpatient dictation letters, radiographs, operation notes and theatre records, and outpatient clinical letters were scrutinised as well as any evidence and cause for subsequent admission to our hospital for all identified patients meeting the inclusion criteria. The Müller AO/Orthopaedic Trauma Association fracture classification and Unified Classification system (UCS) were used to classify native femoral and periprosthetic femoral fractures respectively.^13,14^ In instances where classification was not documented on consultant clinical letters or on the operation note, radiographs were reviewed and assessed. All timings were calculated with respect to admission at the treating centre’s Emergency Department. NHFD data for hip fracture patients admitted to our unit during the same time period was obtained following necessary information governance approvals, enabling comparison of patient cohorts.^15^ Data were collated and unit of analysis was per patient. Results are presented using a combination of descriptive and inferential statistics.

### Statistical methods

Categorical variables were constructed into contingency tables, percentage frequencies calculated and compared with chi-square tests. The data distribution of continuous data was assessed with a D’Agostino and Pearson normality test. Continuous data variables were not normally distributed and therefore groups described with median and interquartile range then compared with Kruskal-Wallis tests and multiple comparisons performed with Dunn’s correction. Survivorship was determined at 30 days, 120 days and 365 days post-admission. Survivorship analysis was performed using Kaplan-Meier estimates for the 3 groups with calculation of 95% confidence intervals (CIs). Time to event was expressed in months. Survivorship outcomes between groups were compared using a Cox proportional hazards model. Statistical significance was set at *p* < 0.05.

## Results

### Patient characteristics

There were a total of 145 femoral fractures identified which met the inclusion criteria featuring 144 patients (1 patient experienced another fracture at a later date). This comprised 58 native bone femoral fractures and 87 periprosthetic femoral fractures. Over the same time period, there were 1032 hip fractures. This equates to a relative incidence of approximately 6 native bone femoral fractures and 9 periprosthetic femoral fractures for every 100 hip fractures.

The majority of patients were female (70%) and the most common American Society of Anesthesiologists (ASA) grade was 3 for all patient cohorts with no significant differences observed between the groups (chi-square; *p* *=* 0.924 and *p* *=* 0.081 respectively). Patients sustaining native bone femoral fractures were found to be significantly younger than hip fracture patients at the time of their injury; median age 77 years (interquartile range [IQR] 70–86) versus 84 years (IQR 76–89) (Kruskal-Wallis; *p* *=* 0.007). However, there were no significant differences in age between patients sustaining periprosthetic femoral fractures compared to hip fractures and native bone femoral factures (Kruskal-Wallis; *p* *=* 0.999 and *p* *=* 0.218 respectively).

With regards to patient’s residence and mobility statuses prior to injury, there were significant differences observed between the 3 patient cohorts. A relatively higher proportion of periprosthetic femoral fracture patients lived in their own home (92% vs. 86% and 81% for native femoral and hip fracture patients respectively) (chi-square; *p* *=* 0.044) and a relatively greater proportion of native bone femoral fracture patients were independently mobile or used one aid (63% vs. 43% and 56% for hip and periprosthetic fracture patients respectively) (chi-square; *p* *=* 0.002). Furthermore, the greatest proportion of those able to mobilise outside their own home only with assistance was seen in the hip fracture group (48% vs. 12% and 10% in native femoral and periprosthetic fracture patients respectively) (chi-squared; *p* *<* 0.001). Patient characteristics of all 3 cohorts are detailed in Table 1. Supplemental Table 2 illustrates breakdown by type of periprosthetic femoral fracture.

**Table 1.** Patient characteristics and injury details.

		Native Femoral Fractures	Periprosthetic Femoral Fractures	Hip Fractures
Fractures (n)		58	87	1032
Median age (years)		77	82	84
Interquartile range (years)		70–86	75–89	76–89
Gender	Male	16 (31%)	26 (30%)	310 (30%)
	Female	42 (69%)	61 (70%)	722 (70%)
ASA grade	I	3 (5%)	1 (1%)	11 (1%)
	II	12 (21%)	26 (30%)	249 (24%)
	III	26 (45%)	45 (52%)	562 (55%)
	IV	17 (29%)	15 (17%)	189 (19%)
	V	0 (0%)	0 (0%)	6 (1%)
Residence before admission	Own home/sheltered housing	48 (86%)	80 (92%)	831 (81%)
	Residential care	6 (11%)	2 (3%)	99 (10%)
	Nursing care	2 (4%)	5 (6%)	102 (10%)
Pre-fracture mobility	Independent	27 (48%)^a^	25 (29%)	301 (29%)
	1 aid	8 (14%)	24 (28%)	147 (14%)
	2 aids/ZF	6 (10%)	25 (29%)	71 (7%)
	Outdoors with assistance only	7 (13%)	9 (10%)	499 (48%)
	No functional mobility	8 (14%)	4 (5%)	14 (1%)
Side injured	Left	30 (52%)	45 (52%)	527 (51%)
	Right	25 (43%)	41 (47%)	505 (49%)
	Bilateral	3 (5%)	1 (1%)	0 (0%)
Open fracture (*n*)		8 (14%)	3 (3%)	^b^
Number of injuries and level of trauma	Isolated	39 (67%)	76 (87%)	^b^
	Additional injury	3 (5%)	5 (6%)	
	Polytrauma	16 (28%)	6 (7%)	
Admission type	Via ED§	57 (98%)	63 (72%)	982 (95%)
	Secondary transfer	0 (0%)	18 (21%)	9 (1%)
	Inpatient	1 (2%)	6 (7%)	41 (4%)

ASA, American Society of Anesthesiologists; ZF, Zimmer frame; ED, Emergency Department.

a 2 patients had missing data.

b Outcome not included in NHFD dataset.

### Injury details

The majority of injuries in the entire study cohort were isolated. A higher proportion of native femoral fractures than periprosthetic femoral fractures were in patients sustaining polytrauma (28% vs. 7%) (chi-square; *p* *=* 0.001). There were no significant differences observed regarding the side of injury (chi-square; *p* *=* 0.939).

Significant differences were found in the route of admission or patient location at the time of injury (chi-square; *p* *<* 0.001). Over 95% of patents with hip fractures and native femoral fractures were admitted *via* the Emergency Department of the treating centre whereas 21% of patients with periprosthetic fractures were secondary transfers. Injury details are included in Table 1.

### Treatment of native bone femoral fractures

**Figure 1.**
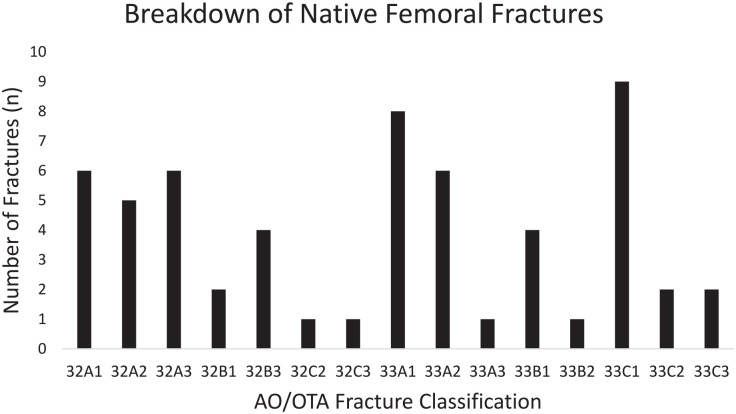
Breakdown of native bone femoral fractures according to Müller AO/Orthopaedic Trauma Association fracture classification.

The majority of fractures occurred in the distal femur (33/58; 57%) and the most common injury was AO 33C1 (16%). A breakdown of fracture classification is shown in Figure 1. The majority of femoral fracture patients were operatively managed (46/58; 79%) and similarly, a minority of hip fracture patients were managed conservatively (2%). Intramedullary nailing in isolation accounted for 25/46 (43%) of all procedures performed for the femoral fracture patients. Other treatments included open reduction internal fixation (ORIF) (24%), distal femoral replacement (5%), intramedullary nail and ORIF (3%), minimally invasive plate osteosynthesis (2%), amputation (2%) and conservative management (21%). Significantly fewer femoral fracture patients received an orthogeriatrician or medical assessment during admission compared to hip fracture patients (41.1% vs. 98.6%; chi-square *p* *<* 0.0001). However, time to orthogeriatrician assessment of the patient was significantly earlier in the femoral fracture cohort compared to the hip fracture cohort for the subset of patients who received a review (median time 9.5 hours (IQR 1.0–30.0) compared to 21.9 hours (IQR 15.4–44.0); Kruskal-Wallis *p* *=* 0.0003). The median time to surgery was 22.2 hours (IQR 16.6–38.3) for the native femoral fracture group and 21.6 hours (IQR 16.7–29.5) for the hip fracture group. However, 74% of operatively managed native femoral fracture patients received surgery within 36 hours of admission compared to 80% of hip fracture patients. Causes for delays to theatre beyond 36 hours for native femoral fracture patients are shown in Table 2.

**Table 2.** Causes for surgical delay exceeding 36 hours from admission in the native femoral fracture and periprosthetic femoral fracture patient cohorts.

Cause	Native femoral fractures *N* = 12 *n* (%)	Periprosthetic femoral fractures *N* = 42 *n* (%)
Awaiting surgeon	2 (15%)	21 (48%)
Trauma load	2 (15%)	2 (5%)
Patient optimisation	2 (15%)^a^	1 (2%)
Clinical decision-making process	1 (8%)^b^	1 (2%)
Unknown^c^	5 (39%)	8 (18%)
Preceding procedure required		3 (7%)
Trial of conservative management		2 (5%)
Equipment related		2 (5%)
Anticoagulation		1 (2%)
Further imaging needed		1 (2%)

a 1 patient was being managed for a head injury, and another patient being treated for a lower respiratory tract infection and deep vein thrombosis.

b 1 patient required an oncological opinion prior to their procedure.

c Not documented in notes.

### Treatment of periprosthetic femoral fractures

**Figure 2.**
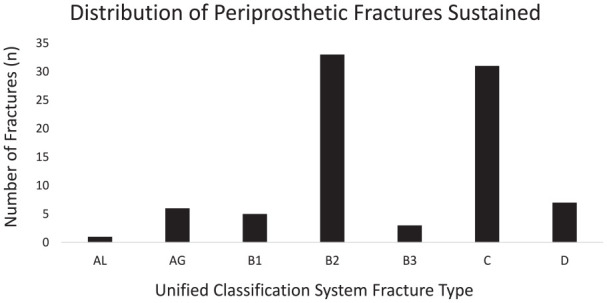
Breakdown of periprosthetic femoral fractures according to Unified Classification System (A – trochanteric fracture, B – around bed of implant, C – distal to implant, D – in the bone between 2 implants).

The most common fracture pattern was UCS B2 (38%) followed closely by UCS C (36%). A breakdown of fracture classification is shown in Figure 2. Surgical management was undertaken for 76/87 (89%) of all cases. Treatments included revision arthroplasty (41%), ORIF (37%), intramedullary nailing (4%), Dall Miles cabling (2%), amputation (1%), Girdlestone procedure (1%) and conservative management (13%). Revision arthroplasty was performed in 50% and 26% of patients with fractures around their hip and knee prosthesis respectively. In contrast, ORIF accounted for 47% and 28% of all treatments in patients with fractures around their knee and hip prosthesis respectively (Supplemental Table 2). There were significant differences between the number of periprosthetic femoral fracture and hip fracture patients who received an orthogeriatrician review during admission (54.7% vs. 98.6% respectively; chi-square; *p* *<* 0.0001) although median time to review between these 2 groups for those that were reviewed were not significantly different; 28.5 hours (IQR 17.5–69.0) versus 21.9 hours (IQR 15.4–44.0). Median time to surgery was significantly longer (Kruskal-Wallis; *p* *<* 0.0001) for patients with a periprosthetic femoral fracture than hip fracture; 44.7 hours (IQR 23.4–93.9) versus 21.6 hours (IQR 16.7–29.5). Only 33/76 (43%) of patients underwent surgery for their periprosthetic fracture within 36 hours of admission compared to 80% of hip fracture patients. Furthermore, these figures do not take into account any additional delays incurred prior to admission to our hospital for patients who were secondary transfers. The most common reason for delay to surgery was specialist surgeon availability, and time to surgery was almost double for patients requiring revision arthroplasty compared to combination of ORIF, intramedullary nailing and Dall Miles Cabling (median 64 hours; IQR 20.9–116.3 vs. median 33 hours; IQR 23.7–70.4 respectively). The remaining patients were delayed due to a variety of other reasons including lack of availability of equipment, reversal of anticoagulation, optimisation of critically unwell patients, and requirement for preceding procedure such as spinal stabilisation or joint aspiration to exclude infection (Table 2).

### Rehabilitation

The majority of native and periprosthetic femoral fracture patients were mobilised full weight-bearing postoperatively (63% and 74% respectively). Further details of postoperative weight-bearing status is provided in Supplemental Table 3.

No significant differences were seen in the length of stay (LOS) between patients admitted following a hip fracture (median 16 days; IQR 10–25), native femoral fracture (median 15 days; IQR 9–30) or periprosthetic femoral fracture (median 17 days; IQR 10–35).

### Complications and mortality

**Figure 3.**
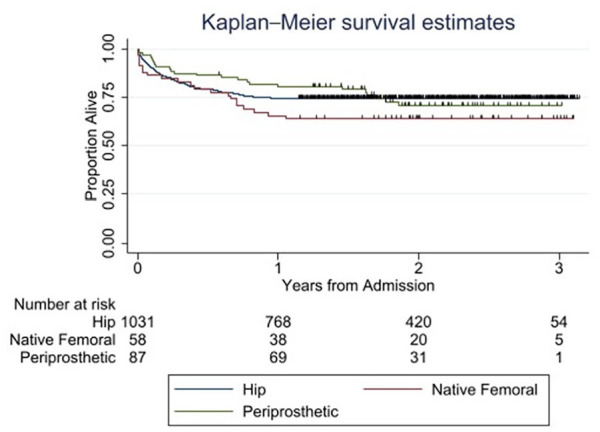
Kaplan-Meier survival curves for the patient cohorts.

The postoperative complication rate was 20/58 (34%) and 36/87 (41%) for native bone and periprosthetic femoral fractures respectively (Tables 3 and 4). In the hip fracture cohort, mortality was 8.7% (95% CI, 7.1–10.6) at 30 days, 18.3% (95% CI, 16.1–20.8) at 120 days and 25.6% (95% CI, 23.0–28.3) at 365 days. In the native femoral fracture cohort, the mortality rates were 13.8% (95% CI, 6.9–25.5), 17.2% (95% CI, 9.4–29.5) and 34.5% (95% CI, 23.2–47.8) respectively and in the periprosthetic fracture cohort, the mortality rates were 3% (95% CI, 1.1–10.3), 11.5% (95% CI, 6.2–20.2) and 19.5% (95% CI, 12.6–29.5) respectively. Exclusion of native femoral fracture patients who sustained polytrauma slightly improved the 30-day mortality to 9%, however, 1-year mortality rate was marginally worse at 36% in that group. Figure 3 shows the Kaplan-Meier survival curves for the 3 patient cohorts. A Cox proportional hazards model containing parameters for patient age (continuous) and fracture type (native vs. periprosthetic vs. hip) revealed a significant difference between native femoral and hip fractures (hazard ratio [HR] 1.75; 95% CI, 1.12–2.73) but not between periprosthetic femoral and hip fractures (HR 1.01; 95% CI, 0.66–1.55).

**Table 3.** Postoperative complications in the native femoral fracture patient cohort.

Complication	Number (*n*)
*Medical*	
Deep vein thrombosis	2
Myocardial infarction	2
Pulmonary embolus	1
Lower respiratory tract infection	1
Further fall	1
*Surgical*	
Knee pain +/- instability	3 (1 revised to TKR)
Delayed union +/- non-union	3 (1 revised)
Deep infection	2
Trendelenburg gait	1
Malunion	1
Non-union	1
Poor mobility	1
Fracture	1
Removal of metalwork	1

TKR, total knee replacement.

**Table 4.** Postoperative complications in the periprosthetic femoral fracture cohort.

Complication	Number (*n*)
*Medical*	
Further fall	4
Deep vein thrombosis	3
Lower respiratory tract infection	2
Cardiac arrhythmia	1
Persisting hypotension requiring ITU transfer	1
Acute kidney injury	1
Bursitis/tendinosis	1
*Surgical*	
Revision procedures	
Prosthetic joint infection	2
Failure of fixation	2
Malreduction	1
Recurrent dislocation	1
Stem subsidence	3
Fracture	2
Deep infection (managed nonoperatively)	2
Thigh pain	2
Trochanteric escape	2
Failure of fixation (managed nonoperatively)	1
Knee pain	1
Stitch collection requiring antibiotics	1
Removal of metalwork for pain	1
Another periprosthetic fracture following discharge	1
Escape of lesser trochanter	1

ITU, Intensive Therapy Unit.

## Discussion

Our study results have shown similarities and differences in the characteristics and management of patients ⩾60 years admitted with a native femoral fracture, periprosthetic femoral fracture, or hip fracture. Most patients were female (70%) and comorbid with an ASA grade ⩾3 for all patient groups. Significant variations were observed in patients’ pre-injury mobility with significantly fewer hip fracture patients able to mobilise freely without aids, and outdoors without assistance, compared to native femoral fracture patients. While most patients in our study were admitted directly to our hospital, a significant proportion of patients with periprosthetic femoral fractures (21%) were secondary transfers. Following admission to hospital, only 41% and 55% of patients with a native femoral fracture and periprosthetic femoral fracture received an orthogeriatrician assessment compared to 98.6% of hip fracture patients. However, native femoral fractures patients were reviewed relatively more quickly than patients in the other groups, and their time to surgery was comparable to hip fracture patients. A possible explanation for this finding may be due to the higher proportion of these patients experiencing polytrauma, admission to Intensive Therapy Unit (ITU), and increased prioritisation of care. Periprosthetic femoral fracture patients experienced the longest time to surgery (median 44.7 hours; IQR 23.4–93.9) with delays most commonly due to lack of surgeon availability. Following surgery, most patients were full weight-bearing and length of stay was not significantly different between patient groups. Patients sustaining native femoral fractures had a higher mortality risk compared to hip fracture patients (HR 1.75; 95% CI, 1.12–2.73), however, no differences were observed between periprosthetic and hip fracture patients (HR 1.01; 95% CI, 0.66–1.55).

There are a limited number of studies that have reviewed the management and outcomes of patients sustaining native or periprosthetic femoral fractures, and even fewer have compared these results to hip fracture patients. Smith et al.^7^ performed a multicentre retrospective study and included patients who sustained native bone or periprosthetic fractures of the distal femur only. They identified similar orthogeriatric review practices to our study although 86% of patients received surgery within 36 hours of their admission. However, most procedures were fracture fixation involving either ORIF using plate and screws (71%) or intramedullary nailing (20%), and revision arthroplasty only accounted for 2% of operations performed. Reasons for the variation in procedures performed compared to our study may be due to differences in study time period, proportion of patients with loose implants, as well as surgeon and patient preferences regarding the benefits and risks of the possible procedures such as postoperative weight-bearing status and non-union.^16^ Mean length of stay was found to be 29 days and mortality rates were 7%, 16% and 18% at 30 days, 6 months and 1 year respectively. A more recent retrospective study by Bommireddy et al.^17^ compared outcomes of patients sustaining closed, unilateral native bone femoral fractures or periprosthetic femoral fractures distal to the prosthesis not requiring revision (i.e. UCS C) to hip fracture patients. Their results demonstrated that mean time from admission to surgery was 1.7 days (range 0–21 days) whereas mean length of stay was slightly longer than in our study (20 days; IQR 12–27.5). Mortality at 30 days and 1 year were reported to be 13.2% and 26.4% respectively. Variations in mortality rates between our results and the aforementioned studies are likely due to several reasons including differences in patient population as well as time to surgery which has been shown to significantly influence 30-day mortality in a meta-analysis of 3 cohort studies.^18^

This is the only study comparing the management and outcomes of patients sustaining the 3 possible types of injury of the femur. Furthermore, in contrast to other studies in the literature, we did not impose restrictions on our inclusion criteria such as level or characteristics of the injury enabling our results to be more pragmatic and reflective of true practice. However, given our hospital is a Major Trauma Centre, our results may not be generalisable to orthopaedic Trauma Units. The use of NHFD patient level data enabled us to perform a more comprehensive comparison for a greater number of outcomes such as timing of surgery, and occurrence and timing of orthogeriatric assessment. However, we were unable to determine whether native femoral fracture and periprosthetic femoral fracture patients received the remainder criteria required to achieve the BPT such as fracture prevention assessment (falls and bone health review), nutritional review, and physiotherapy assessment. This was due to the limitations associated with a retrospective case note review. Nonetheless, based on the available results, only a minority of native femoral fracture and periprosthetic femoral fracture patients received care meeting the full criteria of the BPT in stark contrast to hip fracture patients.^19^ Lastly, whilst mortality rates are likely to be fairly accurate, postoperative complication rates are most likely underestimated. This is particularly the case for polytrauma patients who are typically repatriated and followed up at their local hospital once stable postoperatively.

Surgeon availability was the most cited reason for delay to surgery in the periprosthetic femoral fracture group. Approximately ⅔ of patients who experienced a delay required a revision arthroplasty procedure. Unlike procedures for hip fractures and native bone femoral fractures, revision arthroplasty procedures are performed by a small subset of trauma and orthopaedic surgeons. The incidence of these injuries is projected to rise due to an ageing population and increased demand for arthroplasty procedures.^20–22^ This issue is therefore important to consider for the future planning of the healthcare service and training more surgeons in revision arthroplasty techniques is warranted.

The NHFD has enabled auditing of the care of hip fracture patients facilitating improvements in outcomes, evidenced by consistently reported trends showing lower 30-day patient mortality rates.^15^ Introduction of the BPT initiative in 2012 enabled the standardisation of patient care to defined clinical standards, and has been shown to be associated with significantly increased survival rates at 30 days and 1 year in patients whose care satisfied the BPT criteria compared to care which did not (6% vs. 21%; *p* *<* 0.005 and 28.6% vs. 42%; *p* *<* 0.005, respectively).^5^ This finding is supported by further research which demonstrated significantly increased survival at 30 days and 1 year in patients managed at hospitals that participated in the BPT compared to hospitals which did not.^4,6^ Although less common injuries than hip fractures, native femoral fractures and periprosthetic femoral fractures also render patients unable to weight-bear and susceptible to the risks associated with recumbency. Also, patients in our study shared similar comorbidity profiles suggesting similar levels of frailty. For these reasons, it is plausible that implementation of the BPT to include these patient groups could potentially improve this inequality and patient outcomes in a similar fashion to that shown for hip fracture patients. However, this specific question was outside the scope of this study and future research should focus on investigating the effects of standardising and incentivising care for these patient populations. The NHFD has recently extended to include patients aged ⩾60 years sustaining native bone and periprosthetic femoral fractures, and the auditing of this data will help to answer this question.^23–25^

## Supplemental Material

sj-docx-2-hpi-10.1177_11207000231198459 – Supplemental material for Fewer native and periprosthetic femoral fracture patients receive an orthogeriatric review and expedited surgery compared to hip fracture patients Supplemental material, sj-docx-2-hpi-10.1177_11207000231198459 for Fewer native and periprosthetic femoral fracture patients receive an orthogeriatric review and expedited surgery compared to hip fracture patients by Muhamed M Farhan-Alanie, Sam C Jonas, Daniel Gallacher, Michael R Whitehouse and Tim JS Chesser in HIP International

sj-docx-3-hpi-10.1177_11207000231198459 – Supplemental material for Fewer native and periprosthetic femoral fracture patients receive an orthogeriatric review and expedited surgery compared to hip fracture patients Supplemental material, sj-docx-3-hpi-10.1177_11207000231198459 for Fewer native and periprosthetic femoral fracture patients receive an orthogeriatric review and expedited surgery compared to hip fracture patients by Muhamed M Farhan-Alanie, Sam C Jonas, Daniel Gallacher, Michael R Whitehouse and Tim JS Chesser in HIP International

sj-pdf-1-hpi-10.1177_11207000231198459 – Supplemental material for Fewer native and periprosthetic femoral fracture patients receive an orthogeriatric review and expedited surgery compared to hip fracture patients Supplemental material, sj-pdf-1-hpi-10.1177_11207000231198459 for Fewer native and periprosthetic femoral fracture patients receive an orthogeriatric review and expedited surgery compared to hip fracture patients by Muhamed M Farhan-Alanie, Sam C Jonas, Daniel Gallacher, Michael R Whitehouse and Tim JS Chesser in HIP International
