# Supplementary material for: Fewer native and periprosthetic femoral fracture patients receive an orthogeriatric review and expedited surgery compared to hip fracture patients
Source: Hip Int. 2023 Sep 18;34(2):281–9. doi: 10.1177/11207000231198459 (PMC10935621; doi:10.1177/11207000231198459)
Supplement: sj-docx-2-hpi-10.1177_11207000231198459 – Supplemental material for Fewer native and periprosthetic femoral fracture patients receive an orthogeriatric review and expedited surgery compared to hip fracture patients [file sj-docx-2-hpi-10.1177_11207000231198459.docx]

|  |  | Hip replacement | Knee replacement | Interprosthetic |
| --- | --- | --- | --- | --- |
| Fractures (n) |  | 60 | 19 | 8 |
| Median age (years) |  | 82.5 | 80.0 | 79.6 |
| Interquartile range (years) |  | 75.8 – 86.6 | 76.1 – 92.6 | 71.1 – 95.2 |
|  |  |  |  |  |
| Gender | Male | 41 (68%) | 13 (68%) | 7 (88%) |
|  | Female | 19 (32%) | 6 (32%) | 1 (13%) |
|  |  |  |  |  |
| ASA* grade | I | 1 (2%) | 0 | 0 |
|  | II | 19 (32%) | 4 (21%) | 3 (38%) |
|  | III | 34 (57%) | 7 (37%) | 4 (50%) |
|  | IV | 6 (10%) | 8 (42%) | 1 (13%) |
|  | V | 0 | 0 | 0 |
| Residence before admission | Own home/sheltered housing  Residential care  Nursing care | 58 (97%)  0  2 (3%) | 15 (79%)  2 (11%)  2 (11%) | 7 (88%)  0  1 (13%) |
| Pre-fracture mobility | Independent  1 aid  2 aids/ZF†  Outdoors with assistance only  No functional mobility | 19 (32%)  15 (25%)  16 (27%)  7 (12%)  3 (5%) | 4 (21%)  6 (32%)  8 (42%)  1 (5%)  0 | 2 (25%)  3 (38%)  1 (13%)  1 (13%)  1 (13%) |
| Side injured | Left | 30 (50%) | 10 (53%) | 5 (63%) |
|  | Right | 30 (50%) | 9 (47%) | 3 (38%) |
|  |  |  |  |  |
| Open fracture (n) |  | 1 (2%) | 2 (11%) | 0 |
| Number of injuries and level of trauma | Isolated | 54 (90%) | 14 (74%) | 8 (100%) |
|  | Additional injury | 3 (5%) | 2 (11%) | 0 |
|  | Polytrauma | 3 (5%) | 3 (16%) | 0 |
|  |  |  |  |  |
| Admission type | Via ED‡ Secondary transfer Inpatient | 4 (7%)  13 (22%)  43 (72%) | 0  4 (22%)  15 (79%) | 2 (25%)  1 (13%)  5 (63%) |
| Management | Revision Arthroplasty  ORIF  Nailing  Dall Miles Cabling  Amputation  Girdlestone  Conservative | 30 (50%)  17 (28%)  1 (2%)  2 (3%)  0  1 (2%)  8 (13%) | 5 (26%)  9 (47%)  2 (11%)  0  1 (5%)  0  2 (11%) | 0  8 (100%)  0  0  0  0  0 |

**Supplementary table 2 –** Patient characteristics, injury details, and management by type of periprosthetic femoral fracture
*American Society of Anesthesiologists †Zimmer frame ‡Emergency Department
