# Supplementary material for: Fewer native and periprosthetic femoral fracture patients receive an orthogeriatric review and expedited surgery compared to hip fracture patients
Source: Hip Int. 2023 Sep 18;34(2):281–9. doi: 10.1177/11207000231198459 (PMC10935621; doi:10.1177/11207000231198459)
Supplement: sj-docx-3-hpi-10.1177_11207000231198459 – Supplemental material for Fewer native and periprosthetic femoral fracture patients receive an orthogeriatric review and expedited surgery compared to hip fracture patients [file sj-docx-3-hpi-10.1177_11207000231198459.docx]

|  | Native Femoral Fractures | Periprosthetic Femoral Fractures | Hip  Fractures |
| --- | --- | --- | --- |
| Full weight bearing | 40 (63%) | 65 (74%) | * |
| Partial weight bearing | 5 (8%) | 6 (7%) |  |
| Touch weight bearing | 5 (8%) | 12 (14%) |  |
| Non weight bearing | 4 (6%) | 4 (5%) |  |
| Transfers only | 1 (2%) | 0 (0%) |  |
| Not recorded | 8 (13%) | 1 (1%) |  |
|  |  |  |  |

**Supplementary table 2 –** Comparison of post-operative weight bearing status between patient groups
*Majority of hip fracture cohort patients are allowed to fully weight bear ^16, 17^

16. Bukata SV, Digiovanni BF, Friedman SM, et al. A guide to improving the care of patients with fragility fractures. Geriatr Orthop Surg Rehabil 2011; 2: 5–37.

17. Richardson C, Bretherton CP, Raza M, et al. The Fragility Fracture Postoperative Mobilisation multicentre audit: the reality of weightbearing practices following operations for lower limb fragility fractures. Bone Joint J 2022; 104-B: 972–979.
