# Supplementary material for: Fewer native and periprosthetic femoral fracture patients receive an orthogeriatric review and expedited surgery compared to hip fracture patients
Source: Hip Int. 2023 Sep 18;34(2):281–9. doi: 10.1177/11207000231198459 (PMC10935621; doi:10.1177/11207000231198459)
Supplement: sj-pdf-1-hpi-10.1177_11207000231198459 – Supplemental material for Fewer native and periprosthetic femoral fracture patients receive an orthogeriatric review and expedited surgery compared to hip fracture patients [file sj-pdf-1-hpi-10.1177_11207000231198459.pdf]

- Age at injury
- Gender
- Comorbidities
- American Society of Anesthesiologists (ASA) grade
- Previous arthroplasty procedures (if applicable)
- Pre-fracture mobility
- Residence before admission
- Side injured
- Open/Closed injury
- Fracture classification
- Additional injuries

- Date and time of:
    - Admission to Southmead hospital
    - Orthogeriatric review*
    - Surgery
    - Discharge from Southmead hospital
- Operative technique
- Length of surgery
- Post-operative weight-bearing status
- Reasons for surgical delay
- Medical and surgical complications
- Mortality at 30 days, 120 days and one year

Supplementary table 1 – Data collection parameters
* Orthogeriatrician review was considered to have been performed in one and four hours for patients admitted to the intensive care department and under the care of a medical specialty respectively
